# Ex Situ Examination of Matrix and Inclusions of API-X100 before and after Exposure to Bitumen at Elevated Temperature

**DOI:** 10.3390/ma14175007

**Published:** 2021-09-02

**Authors:** Hongxing Liang, Edouard Asselin

**Affiliations:** Department of Materials Engineering, The University of British Columbia, Vancouver, BC V6T 1Z4, Canada; edouard.asselin@ubc.ca

**Keywords:** API-X100 pipeline, bitumen, corrosion, inclusion

## Abstract

The corrosivity of bitumen at 60 and 120 °C was examined by exposing American Petroleum Institute (API) X100 (yield strength 100 ksi, 690 MPa) pipeline steel to bitumen in an autoclave for 30 days. Prior to the autoclave measurements, the inclusion types in the API-X100 pipeline steel were characterized by scanning electron microscopy (SEM), and four types of inclusions were identified, according to their elemental compositions. The four types of inclusions and the surrounding matrix were characterized by ex situ SEM before and after exposure to bitumen. The results show that no obvious corrosion occured at the inclusions or the matrix after exposure at 60 and 120 °C.

## 1. Introduction

According to the National Energy Board of Canada, by 2040, oil sands bitumen production will reach 4.5 million barrels per day (MMb/d), which is 2.3 times the 2012 production level [1]. By 2040, an increase in bitumen production is also predicted to occur in Venezula (from 0.6 to 2.1 MMb/d), Madagascar (from 0.03 to 0.38 MMb/d), the United States (from 0.005 to 0.035 MMb/d), and the Republic of Congo [2]. Bitumen is typically diluted with a diluent (a natural-gas condensate, such as naphtha) to reduce its viscosity: in this form, it is commonly referred to as “dilbit”. The reduced viscosity enables transportation in pipelines, which is cost effective at large scales [3]. The capacity of existing pipelines is limited, and the construction of new pipelines has been proposed to transport the increasing bitumen production to domestic markets in Canada or new international markets, such as Asia [4]. However, new pipeline proposals result in wide-ranging policy disputes involving both key stakeholders and the general population [5]. For example, the Northern Gateway Pipeline, which planned to ship dilbit from Alberta to British Columbia, was cancelled in 2016 [6]. Similarly, after years of changing status and dispute, the Keystone XL project was cancelled in 2021 [7]. Dusyk et al. [8] found that the dispute was primarily framed as an issue of economic benefit versus environmental risk through the analysis of 2097 articles about the Northern Gateway Pipeline between 2008 and 2014. Among the different environmental risks, terrestrial and aquatic spills of dilbit were common concerns. Dilbit spills resulting from pipeline ruptures or leaks can be related to pipeline corrosion [9]. An insufficient understanding of corrosion of dilbit pipelines, especially their internal corrosion, exacerbates the debate around new pipeline construction.

Though the roles of chloride droplets and deposits in the internal corrosion of dilbit pipelines were reported in [10] and in our previous studies [10,11,12,13,14], the corrosion effect of bitumen itself on the pipeline has been in dispute. Corrosion has been identified in dilbit pipelines near over-bends where deposits coupled with chloride droplets accumulate on the pipeline surface [10]. In separate studies, we used paraffin-covered or dilbit-covered droplets to simulate the corrosive environment in dilbit pipelines. We identified that the distribution of corrosion penetration under the oil-covered droplet is uneven, with the anode regions showing deeper penetration, due to either lower pH [15], enhanced chloride concentration [13], the possible direct reduction of corrosion products [13] or the presence of silica deposition [11]. Furthermore, as summarized in the reports [9,16] or literature [17], and in contrast to other heavy oils, bitumen may lead to faster pipeline corrosion because of the higher concentration of naphthenic acids, sulfur compounds, and asphaltenes that it contains. However, in [18] it was found that only under high temperatures (230–460 °C) could the naphthenic acid compounds in bitumen be corrosive. Thus, one may not expect these compounds to be corrosive at the normal operating temperatures (45–70 °C) of dilbit pipelines [19]. McIntyre, et al., [20] also concluded that bitumen was not corrosive. Via autoclave corrosion measurements of API-X65 (yield strength 65 ksi, 450 MPa) pipeline steel exposed to dilbit and synbit (a mixture of bitumen and synthetic crude oil) at 65 °C for 14 days, they showed that no apparent pitting corrosion occurred on the specimens. Thus, there are conflicting reports on the corrosive nature of bitumen, and this conflict engenders further conflict at the regulatory and project approval stages. In addition, since corrosion around inclusions can be a concern for many materials, it is of added interest to study the potential for corrosion at these microscopic features. To the best of our knowledge, the stability vis-à-vis corrosion of steel inclusions immersed in bitumen has not yet been studied.

Therefore, in order to evaluate the corrosive nature of bitumen, ex situ SEM characterization was used in this work to evaluate the same area on API-X100 pipeline steel before and after exposure to bitumen at 60 and 120 °C for 30 days. These two temperatures were selected because some dilbit pipelines are operated at 60 °C (the now abandoned Keystone XL pipeline) [19], while some pipelines (for example, a test line at MacKay River, AB, Canada) are designed to transport bitumen without dilution at 120 °C [21]. The objective of this work was to see whether bitumen at 60 or 120 °C would cause the corrosion of either the matrix or the inclusions of API-X100 steel. On the one hand, correctly evaluating the corrosion rate of pipeline steel in bitumen provides information for policy actors, key stakeholders and general citizens, who would determine whether to build new bitumen or dilbit pipelines. On the other hand, such work ensures that operators of existing dilbit pipelines use appropriate information for corrosion mitigation, which can provide environmental and economic benefits.

## 2. Materials and Methods

### 2.1. Materials

**Figure 1:**
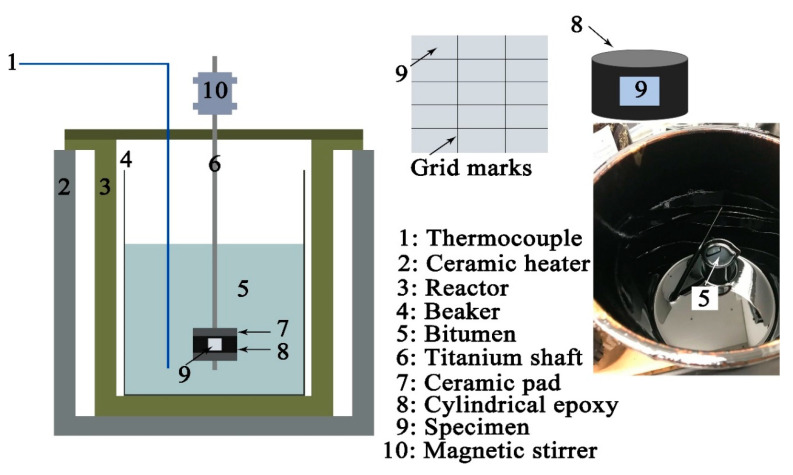
Schematic of the autoclave setup used to examine the corrosion of API-X100 pipeline steel in bitumen at 60 and 120 °C. Bitumen is shown at right in an unrelated separate bucket.

API-X100 (Evraz Inc., Chicago, IL, USA) samples (10 mm × 10 mm × 6 mm in dimension) were cut from a 6 mm thick sheet with a composition provided in Table 1. The specimens were then ground successively, using 120, 320, 600, and 1200 grit silicon carbide papers. After grinding, 6 and 1 µm diamond suspensions were used to polish the steel surfaces. The microstructure of the steel consists of acicular (needle-like) ferrite, polygonal ferrite, and bainite, as reported in our previous study [11]. Grid marks were made on the specimen surface, using a sharp tweezer and ruler without etching in order to locate the same areas by SEM prior to and after autoclave immersion in bitumen, as shown in Figure 1.

### 2.2. Bitumen and Organic Solvent

Bitumen was supplied by a large Canadian producer. The properties and elemental constituents of the bitumen were given in our previous study [13]. Bitumen is a heavy oil with an atomic C/H ratio of 0.72. Importantly, the sulfur concentration in bitumen (4.09 wt.%) is higher than that in crude oil (~3 wt.%) [19]. After the autoclave immersions, the specimens were cleaned using toluene (C_6_H_5_CH_3_, VWR International, Mississauga, ON, Canada).

### 2.3. Autoclave Exposure Experiments

Figure 1 presents the autoclave setup used to assess the potential corrosivity of bitumen. The temperatures were set to either 60 or 120 °C. The rotational speed was controlled at 720 r/min, and the achieved surface velocity (144 cm/s) across the specimen was equal to the flow rate in a real dilbit pipeline [20]. The steel specimen was mounted in the cylindrical epoxy resin (M.G. Chemicals Ltd., Surrey, BC, Canada) with a temperature tolerance of 250 °C. The diameter of the cylindrical epoxy resin was 3.80 cm. After 30 days of exposure, the steel specimen was cleaned as noted above. At least 3 different API-X100 pipeline steel specimens were exposed in the autoclave at each temperature in order to obtain reliable results. 

### 2.4. Surface Morphology

The morphologies of the matrix and inclusions in the steel prior to and after exposure to bitumen for 30 days at 60 and 120 °C were imaged by a field emission SEM (Zeiss Germany, Oberkochen, Germany). SEM equipped with an EDAX Genesis (Zeiss Germany, Oberkochen, Germany) integrated energy dispersive X-ray spectroscopy (EDS) was used to study the elemental distribution of the inclusions. An accelerating voltage of 20 kV was selected to obtain the images of EDS and secondary electrons. At least 3 different positions in the matrix and 50 different inclusions (the identification of inclusions were conducted using SEM EDS area mapping) were examined to ensure reliable results.

## 3. Results

### 3.1. Inclusions in API-X100 Pipeline Steel

**Figure 2:**
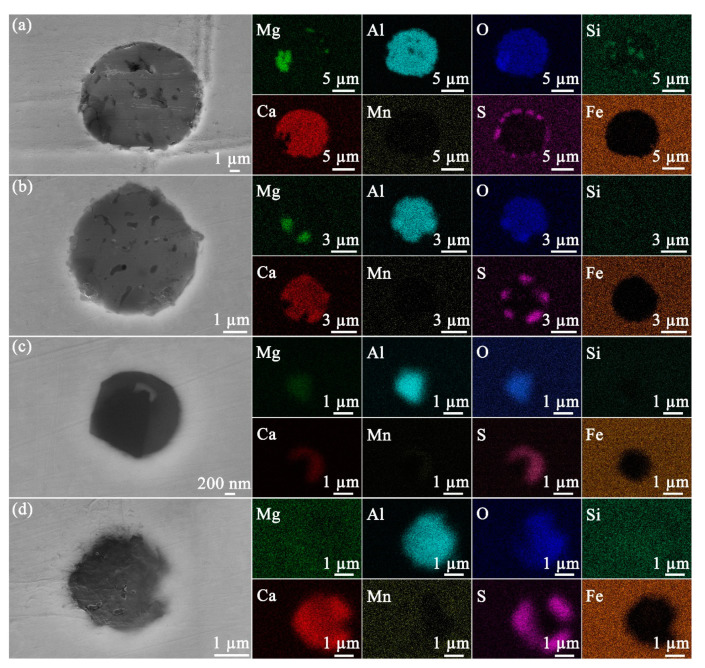
SEM/SE (secondary electron) images of inclusions containing (**a**) Mg−Al−Si−O−Ca−S, (**b**) Mg−Al−O−Ca−S, (**c**) Mg−Al−O−Ca−Mn−S, and (**d**) Al−O−Ca−S on unexposed steel. The elemental distributions of the inclusions are given at right.

At least 50 inclusions randomly selected on the steel surface were observed by microscopy in order to obtain statistically relevant results. Based on the composition measured by EDS, the inclusions were classified into four different types, namely, Mg−Al−Si−O−Ca−S (Figure 2a), Mg−Al−O−Ca−S (Figure 2b), Mg−Al−O−Ca−Mn−S (Figure 2c), and Al−O−Ca−S (Figure 2d). Of the 50 inclusions, these 4 types of inclusions represented 58%, 30%, 10%, and 2% of the total number, respectively. 

### 3.2. Evaluation of the Inclusions after Exposure to Bitumen at 60 and 120 °C

**Figure 3:**
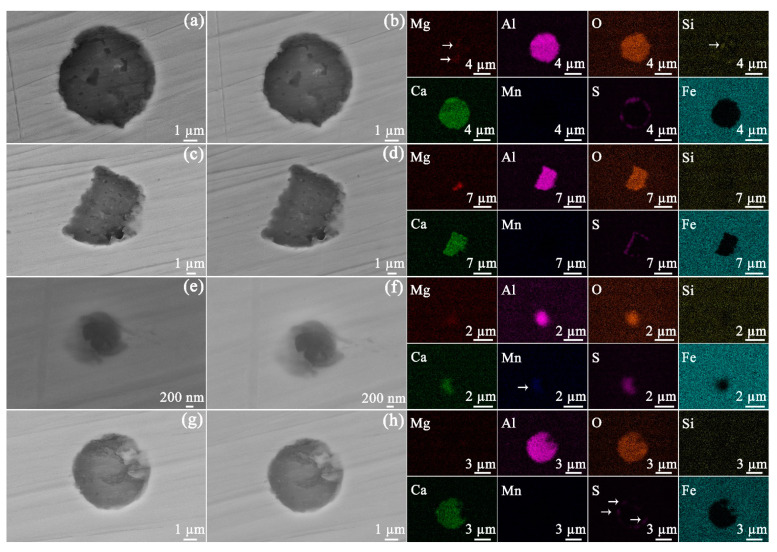
SEM SE images of the inclusion containing Mg−Al−Si−O−Ca−S before (**a**) and after (**b**) exposure to bitumen at 60 °C, of the inclusion containing Mg−Al−O−Ca−S before (**c**) and after (**d**) exposure to bitumen at 60 °C, of the inclusion containing Mg−Al−O−Ca−Mn−S before (**e**) and after (**f**) exposure to bitumen at 60 °C, and of the inclusion containing Al−O−Ca−S before (**g**) and after (**h**) exposure to bitumen at 60 °C. The elemental distributions of the inclusions are given at right. In the EDS maps given at right, the areas with low signal intensity are marked with arrows.

**Figure 4:**
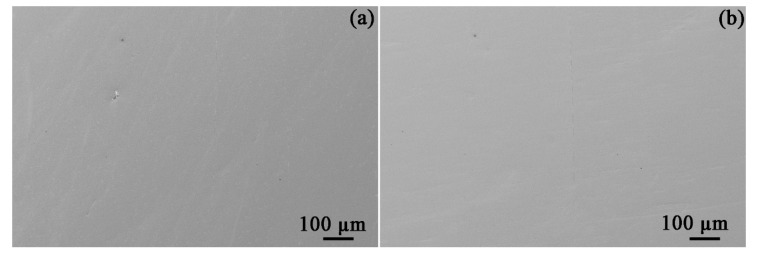
SEM SE images of the API-X100 pipeline steel matrix before (**a**) and after (**b**) exposed to bitumen at 60 °C.

Ex situ SEM characterization of identical areas and inclusions before and after exposure to bitumen avoids providing misleading results. The morphologies of the four types of inclusions prior to and after exposure to bitumen at 60 °C are given in Figure 3. After an exposure of 30 days, no obvious corrosion could be found to occur at the inclusions. Furthermore, the API-X100 pipeline steel matrix (Figure 4) also did not show any signs of corrosion: no trace of uniform or pitting corrosion was detected.

**Figure 5:**
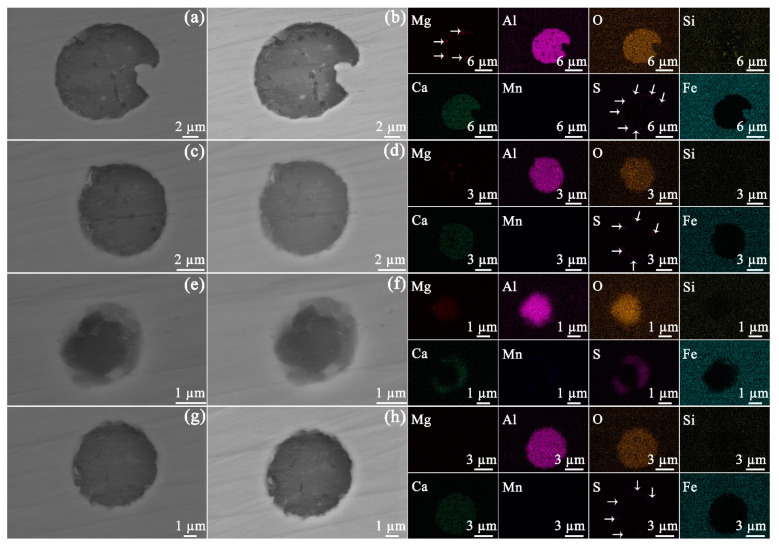
SEM SE images of the inclusion containing Mg−Al−Si−O−Ca−S before (**a**) and after (**b**) exposed to bitumen at 120 °C, of the inclusion containing Mg−Al−O−Ca−S before (**c**) and after (**d**) exposed to bitumen at 120 °C, of the inclusion containing Mg−Al−O−Ca−Mn−S before (**e**) and after (**f**) exposed to bitumen at 120 °C, and of the inclusion containing Al−O−Ca−S before (**g**) and after (**h**) exposed to bitumen at 120 °C. The elemental distributions of the inclusions are given at right. In the EDS maps given at right, the areas with low signal intensity are marked with arrows.

**Figure 6:**
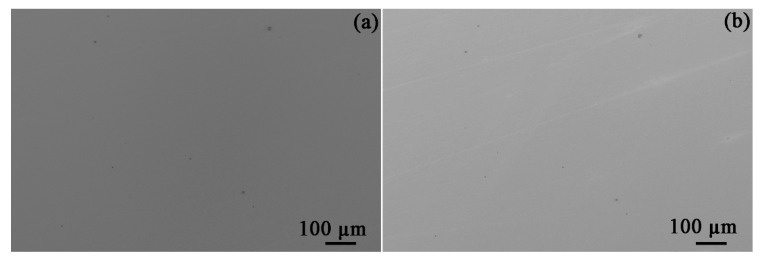
SEM SE images of the API-X100 pipeline steel matrix before (**a**) and after (**b**) exposed to bitumen at 120 °C.

At 120 °C, the four types of inclusions also remained inert after exposure for 30 days (Figure 5). In addition, neither uniform corrosion nor pitting corrosion was observed in the steel matrix (Figure 6). The current results indicate that the bitumen tested here, without diluent, and even at elevated temperature, was not corrosive to API-X100 pipeline steel.

## 4. Discussion

### 4.1. Inclusions in the Steel

In Figure 2, four types of inclusions were identified. The inclusions that are frequently detected in carbon steel include magnesium oxide (MgO), calcium oxide (CaO), silicon dioxide (SiO_2_), aluminum oxide (Al_2_O_3_), calcium sulfide (CaS), and manganese sulfide (MnS) [22]. Further, it is reported that the S in API-X100 is bound in CaS [23]. It is inferred that the inclusion in Figure 2a consists of MgO, Al_2_O_3_, SiO_2_, CaO, and CaS. Compared to the inclusion in Figure 2a, the inclusion of Figure 2b does not show SiO_2_. Further, neither MgO nor SiO_2_ were observed in Figure 2d. As shown in Figure 2c, the Al and Mg are bound with O, whereas the Ca and Mn are bound with S, indicating a separation within the same particle, i.e., into (Ca, Mn)S and (Mg, Al)O rich portions. During refining, the improvement of steel cleanliness and modification of Al_2_O_3_ and MnS are achieved by the addition of Ca [24]. The sulfur in inclusions exists as CaS with the CaS formed by the direct reaction of S and Ca as well as the reaction of CaO with dissolved Al and S in liquid steel [24].

API-X100 pipeline steels from different manufacturers may show different inclusions, which are dependent on the manufacturing, deoxidation, and desulfurization processes [22]. Dong et al. [25] concluded that the primary inclusions in this steel are Al_2_O_3_, titanium dioxide (TiO_2_) and ferric carbide (Fe_3_C). Jin and Cheng reported that four types of inclusions (Si-enriched, Al-enriched, microvoid, and carbide inclusions) were identified in API-X100, and the electrochemical activity of Si-enriched inclusions is higher than that of the matrix in a near-neutral pH bicarbonate solution purged with 5% carbon dioxide (CO_2_) [26]. In another independent study from the same research group, five types of inclusions, including MnS, MnS/Al_2_O_3_, Ca/Al/Mg/O, Al_2_O_3_, and Si-enriched particles were identified in API-X100 [27]. However, Li et al. [28] concluded that the Si-enriched inclusions are inert during the exposure to near neutral pH bicarbonate solution (purged with 5% CO_2_) and that SiO_2_, Al–Si–S–Ca–O, Al–Si–Ca–O, and Al–Ca–O inclusions were observed in the steel. Moreover, Li et al. did not observe the MnS inclusion in API-X100 by SEM. Arafin and Szpunar [29] disclosed that the inclusions in API-X100 pipeline steel can be classified into three types (Si-based, Al–Mg–S–Ca–O, and Al–Mg–O). The findings in the current work are different from those studies. Al_2_O_3_, CaO, and CaS were identified in all four types of inclusions (Figure 2) and (Mg, Al)O/(Ca, Mn)S (Figure 2c) was detected instead of MnS/Al_2_O_3_. Neither SiO_2_ nor MgO were detected in the inclusions (Figure 2d). Because the SEM can only observe a limited area, other types of inclusions may exist in the steel samples used here. However, these 4 types of inclusions represent the entire spectrum of the 50 inclusions imaged herein.

### 4.2. After Exposure to Bitumen at 60 and 120 °C

The different inclusions were inert in bitumen for 30 days at 60 and 120 °C. It was reported that the corrosion initiation of API-X100 in a near-neutral pH bicarbonate solution is associated with localized dissolution at or near inclusions [27]. When X70 steel is exposed to near-neutral pH bicarbonate solution, (Ca, Mn)S particles, which have a lower resistance to corrosion in comparison with the matrix and (Mg, Al)O particles, can dissolve preferentially [30]. However, in bitumen, the CaMnS particles within the API-X100 remained inactive at both temperatures (Figure 3e,f and Figure 5e,f) even after 30 days.

The API-X100 steel matrix also remained inactive (Figure 4 and Figure 6) in the bitumen. Derungs [31] indicated that the corrosion caused by naphthenic acids cannot occur below 220 °C. The sulfur-containing compounds can be separated into two categories, namely, polar (mercaptans) and non-polar (sulfide, thiophenes) forms [32]. Ayello et al. [32] confirmed that 1-tetradecanethiol (a mercaptan) can inhibit the corrosion of carbon steel in chloride solution purged by CO_2_ at room temperature, whereas dibenzothiophene (a sulfide) and dioctyl-sulfide (a thiophene) have no significant effect on carbon steel corrosion. Morales et al. [33] reported that asphaltene can deposit on a pipeline surface and behave as a physical barrier that prohibits corrosive species from contacting the pipeline surface. If a paraffinic solvent diluent is used with bitumen, asphaltene deposition may occur coupled with the accumulation of solid deposits, forming a sludge, which can result in the corrosion of pipelines. However, the main objective of this work was to verify the corrosivity of bitumen without consideration of the diluents. 

Although bitumen itself does not trigger obvious corrosion of pipelines, that does not indicate that the transportation of dilbit by pipelines is absolutely safe. As mentioned in the introduction, corrosion of pipelines can occur under deposits that may contain aqueous chloride droplets. It is still necessary to improve the understanding of dilbit pipeline corrosion, especially its internal corrosion, and to consider other factors, such as microbially induced corrosion.

## 5. Conclusions

In this study, four types of inclusions (Mg−Al−Si−O−Ca−S, Mg−Al−O−Ca−S, Mg−Al−O−Ca−Mn−S, and Al−O−Ca−S) were detected in the API-X100 pipeline steel based on the elemental composition of the inclusions. The Mg−Al−Si−O−Ca−S inclusions consist of MgO, Al_2_O_3_, SiO_2_, CaO, and CaS, whereas SiO_2_ is not observed in Mg−Al−O−Ca−S. The Ca and Mn in Mg−Al−O−Ca−Mn−S are bound with S, while the Al and Mg are bound with O, demonstrating a separation within the same particle, i.e., into (Ca, Mn)S and (Mg, Al)O. Further, Al−O−Ca−S does not show either MgO or SiO_2_. An ex situ SEM/EDS examination of an identical area on API-X100 pipeline steel before and after exposure to bitumen indicates that neither the matrix nor the inclusions corrode at 60 or 120 °C. Insufficient understanding of the corrosivity of bitumen exacerbates the debate around new pipeline construction. The finding herein of the non-corrosive nature of bitumen in dilbit pipeline conditions provides information to policy makers, key stakeholders and citizens, who determine whether to build new dilbit pipelines.

## Figures and Tables

**Table 1:** Chemical composition (assay from the steel manufacturer) of the API-X100 pipeline steel samples (unit: wt.%).

Element	C	Cu	Mn	V	Cr	Nb	Ni	Al	Mo	Ti
API-X100 (wt.%)	0.1	0.25	1.66	0.003	0.016	0.043	0.13	0.02	0.19	0.02

## Data Availability

All the data is available within the manuscript.
